# Identification of a new cell-penetrating peptide derived from the african swine fever virus CD2v protein

**DOI:** 10.1080/10717544.2021.1909178

**Published:** 2021-05-19

**Authors:** Shunli Yang, Xinming Zhang, Yuying Cao, Shuo Li, Junjun Shao, Shiqi Sun, Huichen Guo, Shuanghui Yin

**Affiliations:** a State Key Laboratory of Veterinary Etiological Biology, National Foot-and-Mouth Disease Reference Laboratory, Lanzhou Veterinary Research Institute Chinese Academy of Agricultural Sciences, Lanzhou, P.R. China; b College of Animal Science, Yangtze University, Jingzhou, P. R. China

**Keywords:** ASFV, CD2v, ((KPCPPP)3, RAAS), cell-penetrating peptide (CPP)

## Abstract

The African swine fever virus (ASFV) is a huge and complex DNA virus that can lead to the acute death of pigs and cause huge losses to the global swine industry. The CD2v protein is a transmembrane protein encoded by the ASFV’s *EP402R* gene, which can effectively inhibit the bystander lymphocyte proliferation in response to mitogens and mediate the absorption of red blood cells to ASFV-infected cells. The CD2v protein contains repetitive amino acid sequences ([KPCPPP]_3_ labeled as RAAS), which is reported as a genetic marker and an epitope. However, the specific biological function of the RAAS is unknown. Here, we have found that the truncated CD2v protein with RAAS can enter Chinese hamster ovary cells, but the truncated CD2v protein without RAAS cannot enter the cells. Also, the RAAS can carry the macromolecular protein EGFP to enter various cells through multiple endocytic processes that are dependent on time, concentration, and location. Besides, the RAAS enter the cells via the macropinocytosis or the clathrin-mediated endocytosis. These results indicate that the RAAS can function as a cell-penetrating peptide that provides a new insight for ASFV research and has potential application value as a tool for drug delivery.

## Introduction

The African swine fever (ASF), which is caused by the ASF virus (ASFV), is a highly fatal infectious disease with mortality rates approaching 100%, leads to catastrophic harm for the swine industry, and threatens food security in outbreaks of countries (Zhao et al., 2019). A total of 24 genotypes of ASFV circulate in swine and lead to complex epidemiology (Zhao et al., 2019). The ASFV genome encodes more than 150 open reading frames, which form a mature ASFV virion with a large, enveloped, and complicated architecture. This architecture makes the development of an efficacious vaccine challenging due to the lack of knowledge for research (Gaudreault & Richt, 2019; Liu et al., 2019). Recently, two ASFV-encoded proteins, CD2v (EP402R) and/or C-type lectin (EP153R), are responsible in part for the serotype-specific cross-protective immunity observed for ASF and that these viral proteins are significant protective antigens for ASF (Burmakina et al., 2019). The CD2v protein is the main component of its outer envelope with a C-terminal that has a repeat sequence. Previous research shows that the repeat sequence is a genetic marker or the antigen epitope, but the specific biological function is unknown (Sanna et al., 2017; Burmakina et al., 2019).

Interestingly, the PCV2 Cap protein has a repeat sequence identified as the cell-penetrating peptide (CPP) (Yu et al., 2018). CPPs are a family of various peptides and typically comprise 5–30 amino acids that can pass through tissue and cell membranes via the energy-dependent or energy-independent mechanisms with no interaction with specific receptors (Bohmova et al., 2018). We have found that the CD2v protein C-terminal repeat sequence (KPCPPP)_3_ (RAAS) is similar to the CPP Polyp3(SAP) sequence (VHLPPP)_3_ (Fernandez-Carneado et al., 2004). Here, we identify that the RAAS is the CPP, which can carry the EGFP entering CHO cells, and the inhibitor experiment confirms that the RAAS enters cells via the clathrin- and the micropinocytosis-mediated endocytoses. At the same time, other inhibitors inhibit the entry of CPP. This research provides new insight into the way ASFV particles enter cells and that the ASFV research can be developed into a tool for drug delivery.

## Materials and methods

### Cell and reagents

CHO, Hela S6, ST (American Type Culture Collection, Virginia, United States) were maintained in Dulbecco’s modified Eagle’s medium (high glucose with l-glutamine, Hyclone) containing 10% fetal bovine serum (Biological Industries), 100 units/ml penicillin, and 100 µg/ml streptomycin (Life Technologies), placed in an incubator maintained at 37 °C and 5% CO_2_ and passaged every two days. The Hoechst 33342 was purchased from Solarbio, Beijing, China. All chemicals, unless otherwise stated, were purchased from Sigma-Aldrich.

### Protein expression, purification, and cellular uptake

All gene fragments were from Synthetic (Engineering, Xian, China) and subcloned in the protein expression vector PET-32a (Life Technologies). Recombinant plasmids were analyzed using DNA sequencing for double strands to confirm that no mutation was introduced in both clones. Correct plasmids were then transformed into BL21 (DE3) competent cells (Vazyme, Nanjing, China) for protein expression. When the A600 of the *Escherichia coli* culture reached 0.6–0.8, the proteins of interest (i.e. EGFP, EGFP + RAAS, RAAS + EGFP, CD2v 231-300 aa+ 6xHis, and CD2v 231-319 aa+ 6xHis) were expressed by adding 0.1 mM isopropyl β-d-Thiogalactoside to the culture for 12 h at 16 °C. The recombinant fusion protein was purified using Ni affinity chromatography. The protein concentration was quantified using the Nanodrop one 2000 (Thermo Fisher Scientific, Massachusetts, United States), after which the purified protein (50 μM) was incubated with CHO in 35 mm glass-bottomed Petri dishes (NEST, Wuxi, China). Finally, cells were washed thrice with PBS to remove unbound proteins and imaged using confocal microscopy.

### Peptide synthesis, labeling, and cellular uptake

The peptides of RAAS (KPCPPPKPCPPPKPCPPP) and HIV TAT protein transduction domain (YGRKKRRQRRR) were synthesized by ChenTai, Inc. (Nanjing, China). All peptides were conjugated with FITC at the NH_2_-terminal ends and further purified via HPLC (> 98%). Lyophilized peptides were stored at −40 °C before use. For cellular uptake assays, each peptide was first dissolved in aseptic water as a stock solution (5 mg/ml) and added to cell cultures at various concentrations. After incubation, cells were washed thrice with PBS to remove unbound peptides. Live images were acquired by confocal microscopy.

### Flow cytometry

Cells were seeded on 6-well plates at a density of 1.0 × 10^6^ cells/well and incubated with FITC-conjugated RAAS at various concentrations (1, 5, 10, 50, and 100 μM for 1 h) and time (10, 30, 60, 180, and 300 min at 50 μM final concentration) in fresh Opti-MEM (Life Technologies) at 37 °C. After incubation, cells were washed with PBS and detached by 0.25% trypsin (Life Technologies) at 37 °C for 2 min. Finally, the Dulbecco’s modified Eagle’s medium containing 10% fetal bovine serum was added to terminate trypsinization. Cells were washed thrice with PBS and finally resuspended in PBS before flow cytometry analysis (BD FACSVerse, BD Biosciences). In each sample, 10 000 cells were collected, and the arbitrary fluorescence intensity of each cell was acquired.

### Confocal laser scanning microscopy

Cells were seeded on 35 mm glass-bottomed Petri dishes at a density of 5 × 10^5^ cells/well and incubated with Opti-MEM containing peptides at the indicated concentration (50 μM) for 1 h at 37 °C to detect the cellular uptake of peptides. Afterward, cells were washed twice with PBS and cultured with fresh medium. Before imaging, the Hoechst 33342 was added (10 μM final concentration in medium). Confocal images were collected using an inverted Nikon Tie microscope attached to the Confocal C2 system (Nikon, Japan) with an EMCCD camera (AndoriXonUltra897). The FITC and the Hoechst were excited using 488 and 405 nm lasers, respectively.

### Statistical analyses

Flow cytometry, mean fluorescence intensity, and the percentage of cells with intracellular fluorescence were analyzed for each group by using the Flow Jo software (BD Biosciences). For confocal images, each cell’s fluorescence intensity was measured and processed using the ImageJ software (National Institutes of Health, Bethesda, MD). All statistical analyses were performed using the GraphPad Prism software (Version 8.4, San Diego, CA, USA). Differences were considered significant for *p* < .05 (*), *p* < .01 (**), and *p* < .001 (***).

## Results and discussion

**Figure 1.**
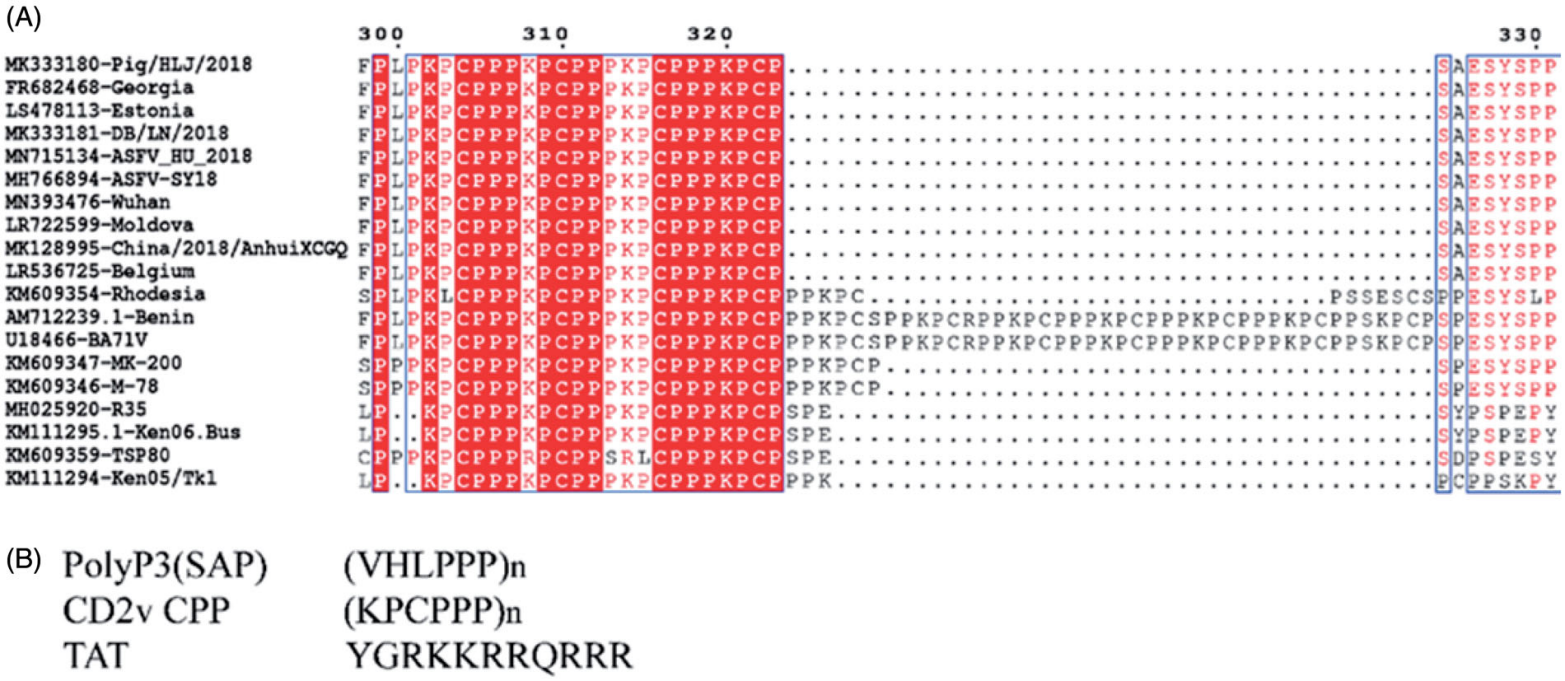
Comparative sequence alignments of amino acid residues. (A) Alignment of the ASFV CD2v amino acid sequence. The CD2v amino acid from 19 ASFV strains isolates collected from the GenBank™ were aligned. The right column’s red amino acid residues indicated strictly conserved residues among all strains. (B) Comparison of the CD2v CPP with two known CPPs.

Our study compared and analyzed the C-terminal region of CD2v (*EP402R* gene) from 19 isolates collected from the GenBank™. A regular feature of the amino acid repeats was found among the reference virus strains, which had (KPCPPP)*_n_* (*n* ≥ 3, Figure 1). Two isolated strains, i.e. AM712239.1-Benin and U18466-BA71V, had the longest repeats of (KPCPPP)_11_, and the residue (hydrophilic amino acid) was substituted by the same chemical property residue, thereby forming a KPCSPP. The isolated ASFV strains of KPCPPP showed the same pattern as the oldest Ca78.2 isolates. ASFV CD2v amino acid sequence analysis revealed some unique amino acid sequence characteristics. The cytoplasmic domain had two clusters of (KPCPPP) proline-rich repeat region in the C-terminal and varied in length among isolates.

**Figure 2.**
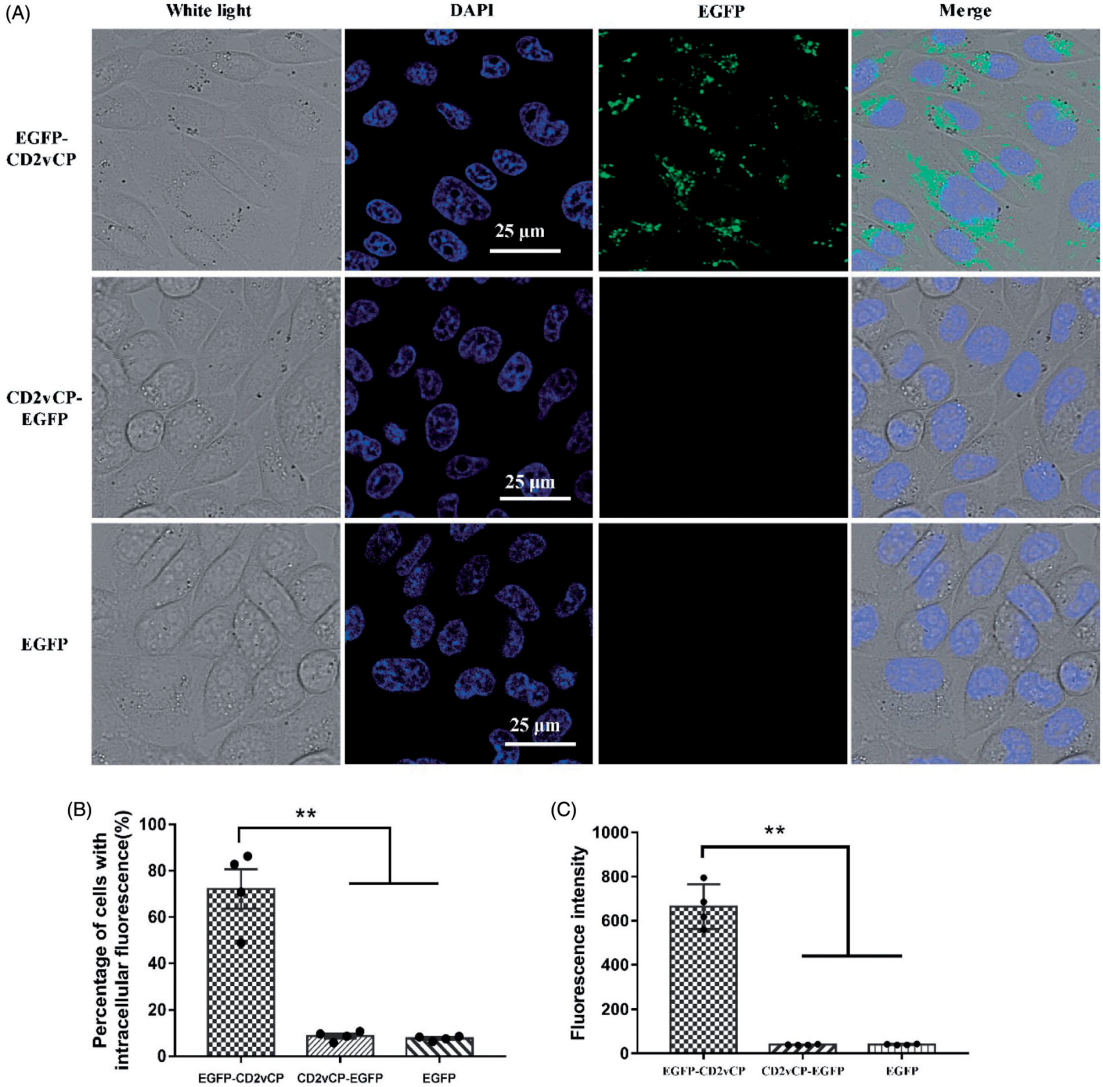
EGFP–linker–RAAS recombinant fusion proteins entered CHO cells. (A) CHO cells were incubated with 50 μg/ml EGFP–linker–RAAS, RAAS–linker–EGFP, and EGFP for 1 h and observed with confocal laser scanning microscopy. The nucleus was stained with Hoechst (blue), and the appearance of a green color meant EGFP signal in CHO cells. (B) Bar graph summarizing the percentage of CHO cells with intracellular fluorescence (*n* = 4; error bars represent S.D. **p < .01). (C) Fluorescence intensity of CHO cells under the above incubation treatments (*n* = 4; error bar represents S.D.; **p < 0.01).

**Figure 3.**
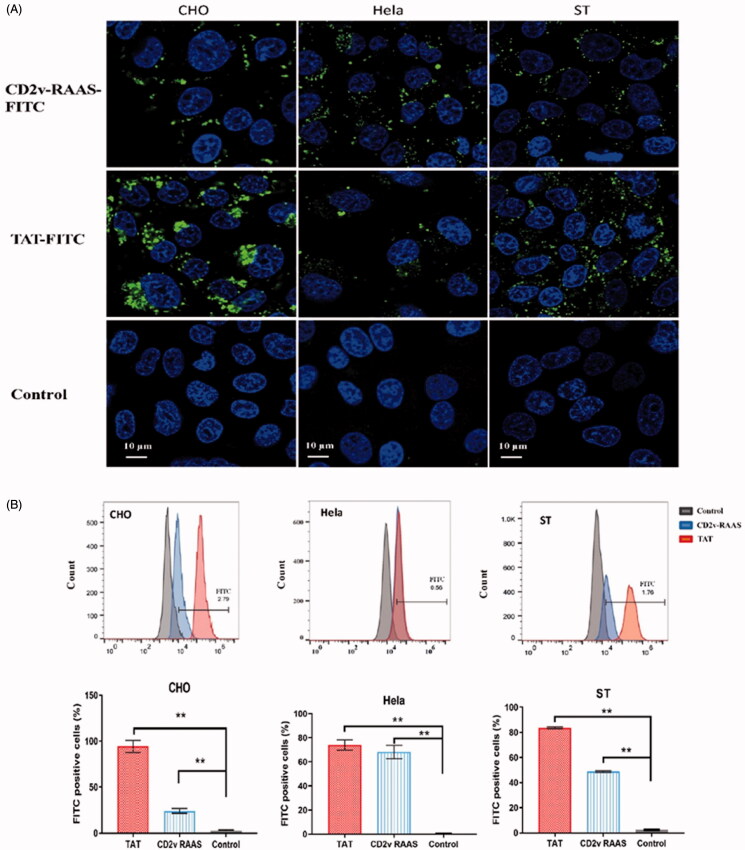
Cellular uptake effectiveness of CD2v RAAS-FITC and TAT-FITC in different cells. (A) CHO, Hela, and ST cells were incubated with 50 μM CD2v RAAS–FITC (upper row) or 50 μM TAT–FITC (middle row) for 1 h and observed with confocal laser scanning microscopy. The control test with cell culture medium only was used as a treatment control (bottom row). The nucleus was stained with Hoechst (blue). PBS was used as a treatment control, and TAT–FITC was used as a positive control. (B) Bar graph summarizing the FITC positive cells(%) of CHO, ST, Hela cells under the above three incubation treatments were analyzed using flow cytometry (n = 3; error bar represents S.D.; **p < 0.01).

**Figure 4.**
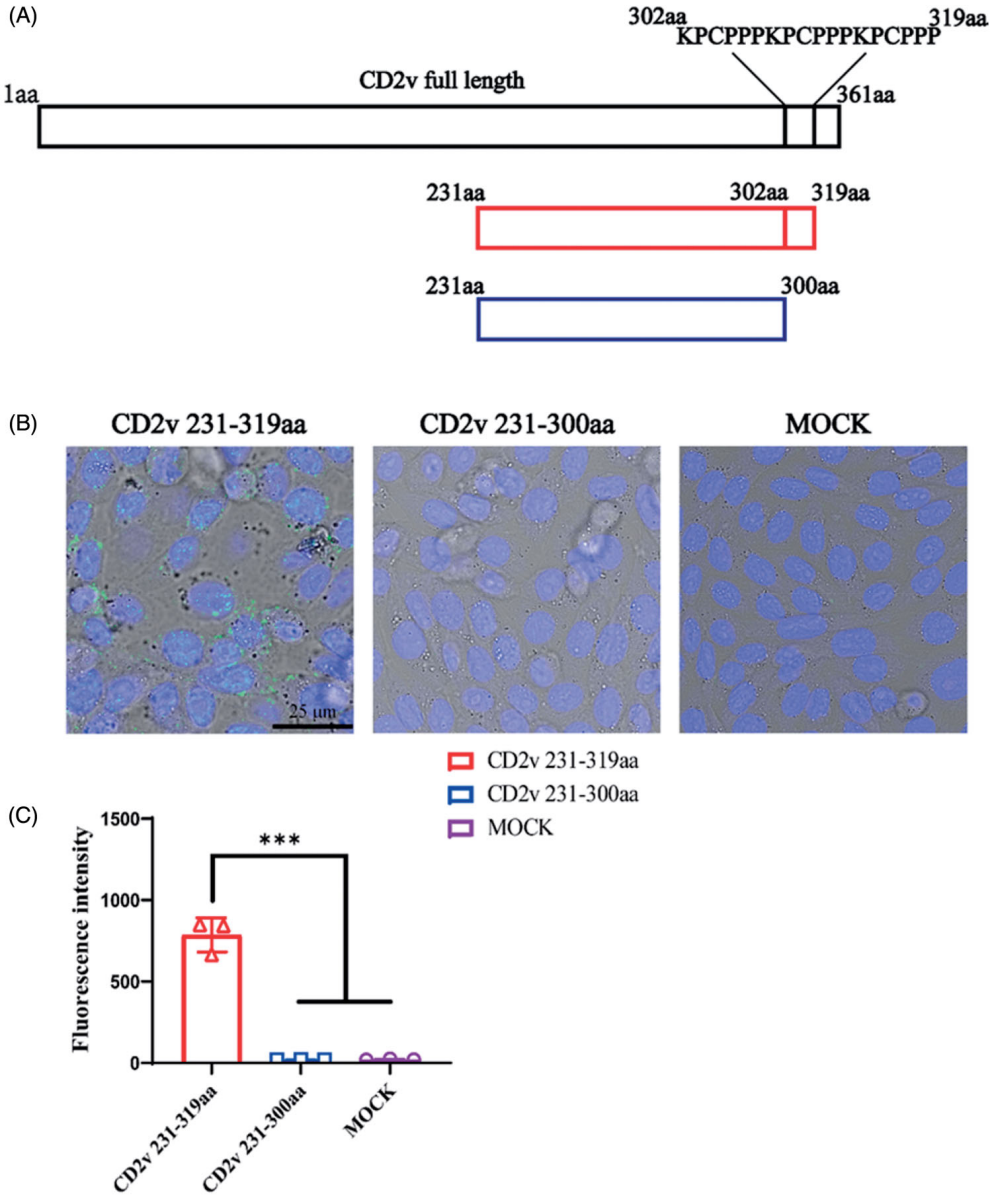
CD2v native protein entered CHO cells. (A) CD2v truncated protein expression pattern. (B) CHO cells were incubated with 50 μM CD2v 231-319 aa and CD2v 231-300 aa proteins at 37 °C and 5% CO_2_ condition for 1 h. After five times washing, truncated CD2v proteins in cells were visualized using anti-His antibody and FITC-conjugated anti-mouse antibody which observed with confocal laser scanning microscopy. The green color means the FITC signal in the CHO cells, and cell nuclei (blue) are indicated by the Hoechst staining. (C) Fluorescence intensity of CHO cells under the above incubation treatments (*n* = 3; error bar represents S.D.; ***p < 0.001).

We postulated that the repeats of (KPCPPP)_n_ (*n* ≥ 3) in the C-terminal of the CD2v function as CPPs. Here, we confirmed the minimum repetitive domain ([KPCPPP]_3_ labeled as RAAS) on the basis of the amino acid sequence comparison (Figure 1). Considering the effective position of RAAS for function, three expression vectors were constructed with pET-32a, i.e. EGFP-linker-RAAS, and RAAS-linker-EGFP, and EGFP. We expressed and purified these three fusion proteins. CHO cells were incubated with purified EGFP-linker-RAAS, RAAS-linker-EGFP, or EGFP (50 μM) for 1 h at 37 °C to determine whether the RAAS of CD2V could carry foreign protein across cell membranes and enter cells *in vitro* (Figure 2). The cell was added to 10 μM Hoechst 33342 to stain the nucleus, incubated for 10 min, and washed with phosphate buffer saline (PBS, pH 7.0). The ImageJ software analysis showed that the RAAS-linker-EGFP or the EGFP of the percentage of cells with intracellular fluorescence lower than EGFP-linker-RAAS was significantly different (four repetitions). We got the tentative conclusion that the position of the RAAS of CD2v affected the CPP crossing the cell membrane. Besides, we presented the synthesis of the RAAS and the TAT peptide labeled with FITC at the C-terminal and examined their ability to cross cell membranes in different cell uptake studies to investigate the penetrating efficiency of the RAAS and the known CPP of the HIV TAT. First, the extent of the uptake of the RAAS-FITC and TAT-FITC at 50 μM for was observed using confocal microscopy (Figure 3). Research results showed that the RAAS-FITC and the TAT-FITC synthesis peptide could enter CHO, Hela, and ST cells. However, the penetrating efficiency of the RAAS-FITC was lower than that of TAT-FITC. Next, we explored whether the truncated CD2v protein could enter the cell. We expressed CD2v truncated proteins with 231–300aa and 231–319 aa fusion His tag (Figure 4(A)). CHO cells were incubated by two CD2v truncated proteins at 37 °C for 1 h and subjected to indirect immunofluorescence assay (Yang et al., 2019). The mouse anti-His (1:1000; Sigma-Aldrich, New Jersey, USA) and the anti-mouse–FITC (1:300; Bioss, Beijing, China) antibodies were used. Confocal laser microscopy results (Figure 4(B)) showed that the 231–319 aa CD2v truncated protein-containing RAAS could enter CHO cells, but the MOCK and 231-300 aa CD2v truncated protein do not containing the RAAS displayed no green fluorescence. This result showed that the native truncated proteins, including the RAAS, could enter the cell through the cell membrane. Besides, flow cytometry showed that the entry of RAAS into cells was dependent on time and dose (Supplementary Figures S1 and S2). CHO cells were pretreated with several inhibitors, i.e. 5 mM methyl-β-cyclodextrin, 50 mM *N*-(ethyl-*N*-isopropyl)-amiloride, 10 μg/ml heparin, and 10 μM Genistein in respective media for 30 min at 37 °C to determine the RAAS entered the cell mechanism. The medium was replaced with a fresh medium containing the RAAS–FITC (50 μM). After 1 h of incubation at 37 °C, cells were washed thrice with PBS and analyzed using flow cytometry (Supplementary Figure S3). Similar to the result of the previous study, the RAAS entered cells through macropinocytosis or clathrin-mediated endocytosis. In conclusion, we identified that the peptides of (KPCPPP)_3_ at the C-terminal in the CD2v protein across the cell membranes was a CPP. This research result could improve the understanding of the ASFV CD2v protein function.

## Supplementary Material

Supplemental Material
